# Next-generation sequencing: A new avenue to understand viral RNA–protein interactions

**DOI:** 10.1016/j.jbc.2022.101924

**Published:** 2022-04-09

**Authors:** Yiyang Zhou, Stephanea L. Sotcheff, Andrew L. Routh

**Affiliations:** 1 Department of Biochemistry and Molecular Biology, The University of Texas Medical Branch, Galveston, Texas, USA; 2 Sealy Center for Structural Biology and Molecular Biophysics, The University of Texas Medical Branch, Galveston, Texas, USA; 3 Institute for Human Infections and Immunity, University of Texas Medical Branch, Galveston, Texas, USA

**Keywords:** next-generation sequencing, crosslinking, viral RNA, RNA-protein interactions, HITS-CLIP, PAR-CLIP, vPAR-CL, human herpesvirus, HIV-1, SARS-CoV-2, CLIP, crosslinking and immunoprecipitation, DMS, dimethyl sulfate, EBV, Epstein–Barr virus, FHV, Flock House virus, HCV, hepatitis C virus, HITS-CLIP, high-throughput sequencing of RNA isolated by crosslinking immunoprecipitation, IAV, influenza A virus, iCLIP, individual nucleotide resolution CLIP, IN, integrase, IP, immunoprecipitation, KSHV, Kaposi's sarcoma-associated herpesvirus, MA, matrix domain, MaP, mutational profile, NC, nucleocapsid, NGS, next-generation sequencing, PAR-CLIP, PhotoActivatable-Ribonucleoside–Enhanced CrossLinking and ImmunoPrecipitation, PIP-seq, Protein Interaction Profile Sequencing, RBP, RNA-binding protein, RIP, RNA immunoprecipitation, RIPiT, RNA:protein immunoprecipitation in tandem, RIP-seq, native RNA immunoprecipitation and sequencing, RNP, ribonucleoprotein, RRE, Rev Response Element, SHAPE, selective 2′-hydroxyl acylation analyzed by primer extension, TAR, trans-activation response, VIR-CLASP, VIRal Crosslinking And Solid-phase Purification, vPAR-CL, viral PhotoActivatable-Ribonucleoside CrossLinking, vRNA, viral RNA, YTHDF, YTH domain family

## Abstract

The genomes of RNA viruses present an astonishing source of both sequence and structural diversity. From intracellular viral RNA-host interfaces to interactions between the RNA genome and structural proteins in virus particles themselves, almost the entire viral lifecycle is accompanied by a myriad of RNA–protein interactions that are required to fulfill their replicative potential. It is therefore important to characterize such rich and dynamic collections of viral RNA–protein interactions to understand virus evolution and their adaptation to their hosts and environment. Recent advances in next-generation sequencing technologies have allowed the characterization of viral RNA–protein interactions, including both transient and conserved interactions, where molecular and structural approaches have fallen short. In this review, we will provide a methodological overview of the high-throughput techniques used to study viral RNA–protein interactions, their biochemical mechanisms, and how they evolved from classical methods as well as one another. We will discuss how different techniques have fueled virus research to characterize how viral RNA and proteins interact, both locally and on a global scale. Finally, we will present examples on how these techniques influence the studies of clinically important pathogens such as HIV-1 and SARS-CoV-2.

RNA viruses and their genomes exhibit incredible sequence and structural diversity and a correspondingly complex range of RNA–protein interactions. Inside infectious virus particles (*in virio*), the specific and often programmed interactions of RNA with viral (nucleo-)capsids orchestrate virus particle assembly, genome packaging and release, structural stability, and can regulate RNA replication and transcription (1, 2, 3, 4). In addition, RNA viruses and their genomes are dependent on host cellular factors and actively reprogram the host cellular environment to support RNA replication and/or prevent immune clearance. Much of this reprogramming occurs through numerous interactions between viral RNAs (vRNAs) and host factors (5). Thus, vRNAs have multiple simultaneous roles and overlapping interactions with both host and viral proteins that must be carefully coordinated during the viral lifecycle. Studying and characterizing this complex choreography presents an important but major technical challenge.

Classical techniques that probe RNA–protein interactions, such as immunoprecipitation (IP) (6, 7), crosslinking (7, 8, 9, 10, 11), EMSA (12, 13), and affinity-capture (14, 15), offer reliable biochemical solutions both *in vitro* and *in vivo*. However, these methods typically rely on prior knowledge or prediction of RNA–protein interaction sites, which requires researchers to target specific hypothesized interactions. This may miss important and/or unanticipated interaction partners. Similarly, due to lack of high-throughput analytical power, molecular methods also are limited in their capacity to identify the many possible RNA targets and binding sites of a protein-of-interest.

The remarkable power of high-throughput next-generation sequencing (NGS) combined with traditional “tried and true” biochemical techniques provides a complementary approach to study vRNA–protein interactions in a high-throughput, specific, and unbiased fashion. This has broad applications in studying the role and activities of vRNA-binding proteins within the host, as well as in elucidating the structure of macromolecular RNA–protein complexes within virus particles themselves. Furthermore, NGS-based methods can often provide nucleotide or near-nucleotide resolution.

As such, NGS is often deployed as a hypothesis-generating instead of hypothesis-dependent tool, allowing for detection of novel RNA–protein interactions within the host transcriptome (*in vivo*) or the viral genomic material (*in virio*). This latter point is particularly important in the case of structural viral proteins, such as icosahedral capsid proteins or helical nucleocapsid (NC) proteins that engage in numerous simultaneous contacts with the viral genomic RNA during viral replication, particle assembly, and disassembly. Details of the molecular contacts of these structural proteins with genomic RNA are often lost in crystallographic or electron-microscopy based approaches due to the use of symmetrical averaging during image reconstruction. These techniques may be appropriate to resolve the symmetrical assemblies of the viral capsid proteins, but they can also obscure the inherently asymmetrically arranged genomic RNA within virus particles. Understanding the unique interactions with the numerous capsid proteins is important, as their relative binding positions and affinities with vRNA must be carefully choreographed to successfully guide virus particle assembly (16). Viral structural proteins can also engage in numerous host RNA contacts. For example, capsid from flaviviruses is not only responsible for wrangling genomic RNA into virus particles but also is shuttled to the nucleus and nucleolus where it mediates diverse, perhaps nonspecific, host–RNA interactions that may be necessary to alter the host transcriptional program to provide a proviral environment (17).

Almost all NGS-based vRNA–protein interaction approaches are derived from or are at least inspired by classical biochemical techniques. In this review, we will discuss how cutting-edge NGS-based technologies evolved from classical methods. We will categorize these NGS approaches based upon whether they employ UV crosslinkers (*e.g.*, HITS-CLIP, PAR-CLIP, vPAR-CL, and etc., Fig. 1), chemical (formaldehyde) crosslinkers (*e.g.*, Protein Interaction Profile Sequencing, PIP-seq), or no crosslinking at all (*e.g.*, native RNA immunoprecipitation and sequencing, RIP-seq), as well as their methods for RNA–protein complex enrichment (IP or affinity capturing). We will also summarize notable applications in virus research (Table 1) and present how each has made an impact on the way we study numerous viruses including SARS-CoV-2 and HIV-1.

**Figure 1:**
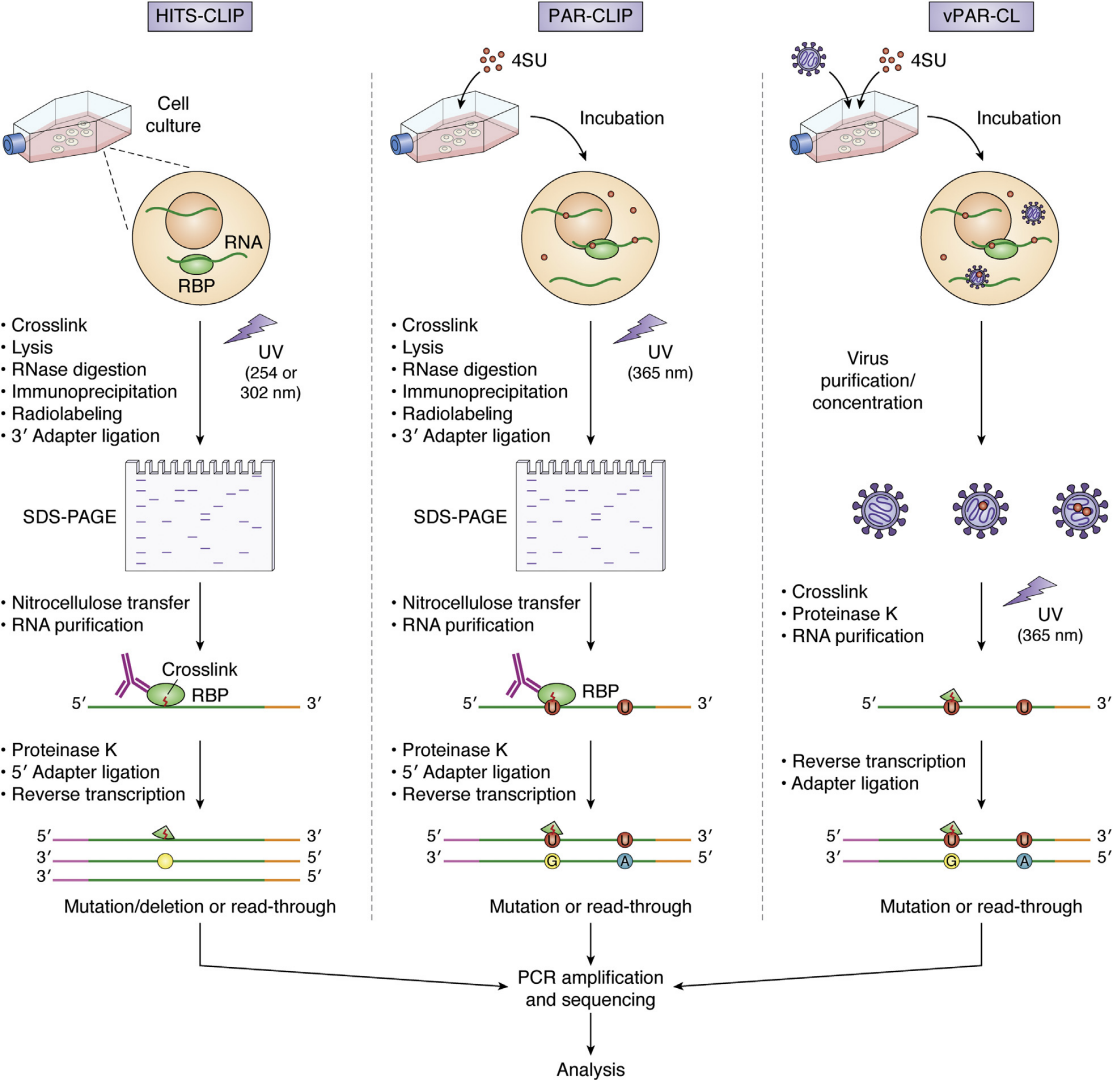
**General methodologies of HITS-CLIP, PAR-CLIP, and vPAR-CL.**

**Table 1:** NGS methods for studying RNA–protein interactions

Methods	Abbreviated procedures	Virus	Reference
Photo-crosslinking methods			
CLIP-seq/HITS-CLIP	UVB/C crosslink, IP, radiolabeling, SDS-PAGE, proteinase K, and NGS	Epstein-Barr virus	(46, 47)
		Kaposi's sarcoma-associated herpesvirus	(42, 69)
		Influenza virus	(54, 55)
		Hepatitis C Virus	(52)
		Simian gammaherpesviruses	(50, 51)
PAR-CLIP	Nucleotide analogs, UVA crosslink, IP, radiolabeling, SDS-PAGE, proteinase K, and NGS	Epstein-Barr virus	(69, 85)
		Kaposi's sarcoma-associated herpesvirus	(69, 85, 86)
		HIV	(89, 90, 102, 106, 111, 112, 114, 121, 122)
		Influenza	(205)
		Moloney leukemia virus 10	(206)
		Herpes Simplex Virus-1	(207, 208)
		Flaviviruses	(209)
		Alphaviruses	(210)
Other CLIP-derived methods	iCLIP	Hepatitis C virus	(211)
		HIV-1 (A3)	(212)
	eCLIP	SARS-CoV-2	(213)
	irCLIP	Flavivirus	(214)
	CLASH	Gammaherpesviruses	(215)
vPAR-CL	Nucleotide analogs, UVA crosslink, proteinase K, NGS	Flock House Virus	(216)
Chemical crosslinking method			
PIP-seq	Formaldehyde crosslink, RNase footprinting, NGS	-	-
Affinity capturing methods			
APEX-seq/Proximity-CLIP	APEX-induced biotinylating, affinity capturing, NGS, and mass spectrometry	SARS-CoV-2	(160)
VIR-CLASP	4SU/photo-crosslinking, solid phase separation, mass spectrometry	CHIKV	(161)
		Influenza A virus	(163)
		Zika	(164)
		SARS-CoV-2	(165)
Non-crosslinking methods			
RIP-seq	Native IP, RNA extraction, NGS	HIV	(178)
		*Bombyx mori* nucleopolyhedrovirus	(181)
		EBV	(182, 183, 184, 217)
		SARS-CoV-2	(180)
Chemical probing	SHAPE/DMS chemicals	HIV-1	(199)

## Photo-crosslinking methods

UV light–mediated crosslinking is a well-established tool for studying RNA–RNA-binding protein (RBP) interactions (18) due to its ability to elicit covalent bonds between adjacent (within covalent-bond distance) amino acid side chains and nitrogenous bases of nucleic acids (19). UV-induced crosslinking provides a means of stabilizing otherwise transient and less stable RNA–protein interactions, which usually consist of hydrogen bonds and electrostatic interactions (19). Zero distance (20) UV-induced RNA-protein crosslinking largely prevents long-distance, nonspecific crosslinks that are commonly induced by chemical crosslinkers such as formaldehyde (21). UV-induced crosslinks are also in general highly specific to nucleic acid–protein interactions.

In both classical biochemical and high-throughput NGS assays, the final analyte is an RNA fragment crosslinked to a protein of interest. UV crosslinking is typically followed by RNase digestion and enrichment of the RBP of interest using approaches such as IP or other affinity-capturing methods. Often, the RBP is digested by proteinase K to leave only small peptide adducts crosslinked to the RNA. Finally, the RNA–peptide complex is analyzed either directly or by reverse transcription into cDNA. Coupled with NGS, a number of high-throughput platforms such as high-throughput sequencing of RNA isolated by crosslinking immunoprecipitation (HITS-CLIP) (22), PhotoActivatable-Ribonucleoside–Enhanced Crosslinking and ImmunoPrecipitation (PAR-CLIP) (23), and viral PhotoActivatable Ribonucleoside CrossLinking (vPAR-CL) (24) have revolutionized the study of vRNA–protein interactions by providing unprecedented sequencing power and scale.

### HITS-CLIP/CLIP-Seq

**Figure 2:**
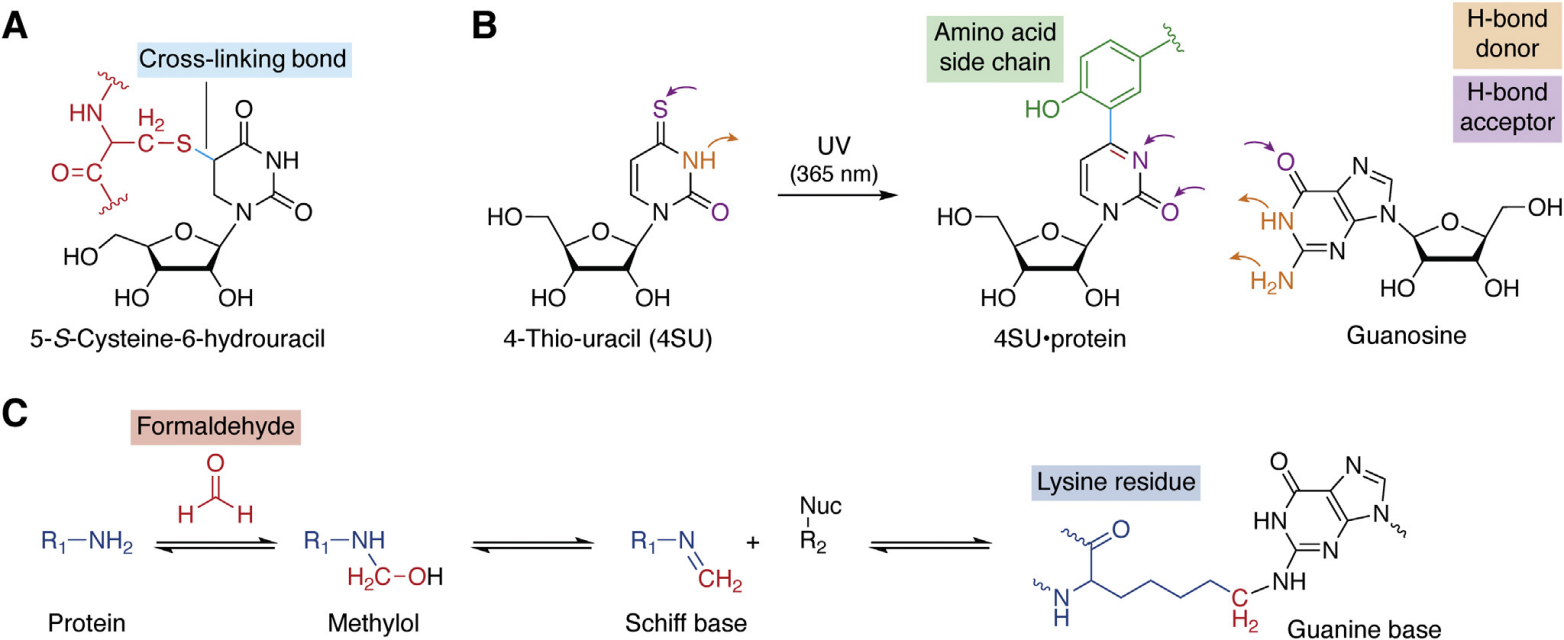
**Mechanisms of common cross-linking methods.***A,* 5-S-cysteine-6-hydrouracil as an example of a cross-link product of 254-nm UV; the *cyan line* indicates the cross-linking bond. Adapted from the study by Smith and Aplin (218). *B,* 4-thio-uracil (4SU) cross-linked with amino acid side chains after 365-nm UV irradiation, which alters hydrogen bond donor/accepter properties of 4SU, and subsequently results in 4SU-guanine mispairing during reverse transcription. *Purple* and *orange arrows* indicate hydrogen bond acceptors and donors, respectively. Adapted from the studies by Hafner *et al.* (23) Ascano *et al.* (80). *C,* molecular mechanism of formaldehyde cross-link: 1) protein; 2) Methylol; 3) Schiff Base; 4) example of lysine–guanine cross-link after formaldehyde cross-link. Adapted from the study by Hoffman *et al.* (21).

CLIP (crosslinking and immunoprecipitation) combines short wavelength UV irradiation and IP to identify RNA sequences interacting with a protein of interest (25, 26). Short wavelength (∼254 nm) UV irradiation has been extensively used to induce RNA-protein crosslinks for nearly half a century (27). In spite of this, the detailed photochemistry and biophysical mechanism of such reaction is not completely understood (28). It is generally accepted (18, 29, 30, 31, 32) that when an aromatic ring (such as the nitrogen-containing aromatic base of nucleotides) is excited by UV irradiation, nucleobases are induced to a higher energetic state to exceed the ionization potential, which generates cation radicals. The consequence of such short-lived high energy nucleobases is either rapid thermal relaxation or the formation of a covalent bond with similar radicals in direct vicinity (such as UV-excited aromatic rings or other side chains in amino acids) (Fig. 2*A*). CLIP was first applied to investigate RNA targets that are bound and regulated by autoimmune neurologic disease antigens (Nova proteins, (33)) in mouse brain tissue (25). After UV crosslinking and anti-Nova IP, CLIP sequence-tags (which represent Nova-binding RNAs) were Sanger-sequenced to reveal RNA motifs associated with alternative spliced mRNAs regulated by Nova (34). These Nova-targeting RNA sites were further validated with Nova^-/-^ mice to demonstrate the advantage of CLIP methodology (25).

In the early days of NGS (35), CLIP quickly benefited and was adapted into HITS-CLIP (22). HITS-CLIP follows the same general scheme of CLIP (22) (Fig. 1): (1) RBP of interest is UV crosslinked with bound RNA *via* short wavelength UV irradiation (typically UVC ∼254 nm or UVB ∼300 nm) in cell culture; (2) cell lysate is treated with RNase to digest unprotected RNA; (3) IP with specific antibody of the RBP of interest enriches the targeted RNA–protein complex; (4) subsequent SDS-PAGE is used to remove noncrosslinked RNA (26); (5) proteinase K is used to cleave the crosslinked protein next to the carboxyl group of hydrophobic or aromatic amino acids (36); (6) the covalent interaction between polypeptide (or sometimes a single amino acid) and ribonucleotide is retained during RNA purification; (7) finally, sequencing adapters/linkers are ligated to the purified RNA–polypeptide complex and RT-PCR is used to reverse transcribe the RNA and generate dsDNA libraries for high-throughput sequencing.

NGS-coupled HITS-CLIP libraries can readily reveal hundreds of thousands of CLIP sequence-tags (22), which comprise RNA fragments ∼50 to 200 nts in length, depending on the sequencing platform used (22, 26). This far exceeds the scale of traditional CLIP (usually only a few thousand CLIP tags (25)). The sequences of CLIP tags are thereafter aligned to genome to determine RBP-binding sites and RNA motifs associated with these interactions (22, 28).

Beyond its original application in mouse brain tissue, numerous applications of HITS-CLIP† have demonstrated its capacity for RNA-RBP discovery on a transcriptomic scale (reviewed in (28)). In virus research, HITS-CLIP shined in the fashion of AGO HITS-CLIP. Argonaute proteins (AGO) are essential members of the cellular RNA-induced silencing complex. Guided *via* their interactions with numerous classes of small noncoding RNAs (such as miRNAs), AGO proteins cleave mRNA and repress translation (37). Due to the small size of AGO-bound miRNAs (∼20 nts.), it can be a challenge to correctly identify their mRNA partners from their sequence alone as the short miRNAs have many potential ambiguous cognate sequences in the transcriptome and miRNAs often bind to their mRNA targets in spite of multiple mismatches. This can be overcome by AGO HITS-CLIP, which demonstrated that AGO protein is sufficiently well associated with miRNAs and mRNAs to allow UV crosslinking of both AGO-miRNA and AGO-mRNA (38). After IP with specific AGO antibody, the discovered miRNA sequences can be used to seed match the codiscovered mRNA targets. AGO HITS-CLIP quickly empowered virus-encoded miRNA discovery in herpesviruses, which encode numerous miRNAs (39, 40). Kaposi's sarcoma-associated herpesvirus (KSHV, also known as human herpesvirus-8) is the tumorigenic cause of Kaposi’s sarcoma (41). KSHV-encoded viral miRNAs are expressed in latently infected cells and are regarded to be associated with viral pathogenesis and tumorigenesis (41). Haecker *et al*. (42) performed AGO HITS-CLIP and recovered thousands of KSHV miRNA targets in KSHV-infected primary effusion lymphoma cells lines, which overlapped with important cellular pathways for KSHV pathogenesis and tumorigenesis. Importantly, AGO HITS-CLIP also recovered significantly different ratios of KSHV miRNAs to human-derived miRNAs, depending on whether the cell lines are postgerminal B cells (BCBL-1, (43)) or pre-B cells (BC-3, (44)). This suggests KSHV infection can potentially compromise the host RNA-induced silencing complex. Similar to KSHV, Epstein–Barr virus (EBV) is another dsDNA herpesvirus (human herpesvirus-4) that persists in humans with latent infection and tumorigenic potential in B cells (45). Riley *et al*. (46) used AGO HITS-CLIP to identify a handful of EBV and human miRNA targets in EBV-transformed B cells (Jijoye cells)and revealed that EBV miRNA predominantly targets human mRNA 3′ UTRs (46). Surprisingly, the highly expressed EBV miRNAs were also found to target human mRNAs that are involved in transcription regulation, apoptosis, cell cycle control, and signaling (46). In a follow-up study, Harold *et al*. (47) further investigated EBV miRNA’s interaction with Caspase protein 3 protein (CASP3). CASP3 is a central host factor regulating apoptosis (48, 49) and has been speculated to be a target of EBV miRNAs for EBV-associated apoptosis repression (46). Using the same AGO HITS-CLIP and more advanced bioinformatics, Harold *et al*. discovered that EBV miRNAs specifically bind to the 3′ UTR of CASP3 mRNA at 13 loci. A subsequent reporter assay confirmed that nine of the discovered EBV miRNAs exhibited significant repression of CASP3, validating the role of EBV miRNAs in targeting CASP3 protein (46). Together, the HITS-CLIP studies on EBV demonstrated the “general-to-specific” approach to understanding viral miRNA functions, in which discovery of protein-binding miRNA targets led to specific hypotheses and experimental validation. Such knowledge can ultimately contribute to defining molecular mechanisms and drug design.

Beside human herpesviruses, HITS-CLIP has been applied in numerous other viruses to study how vRNA and miRNA interact with cellular proteins, such as AGO. With simian gammaherpesviruses (*Herpesvirus saimiri*, HSV), Guo *et al*. used AGO HITS-CLIP to identify the enriched mRNA targets of miR-27 in T-cell receptor signaling pathway (50) and robust AGO-binding sites on both host and HSV genomes that are mediated by viral U-rich miRNAs (51). Luna *et al*. (52) used AGO HITS-CLIP to investigate the relation between hepatitis C virus (HCV) and microRNA-122 (miR-122). miR-122 is a highly expressed liver-specific miRNA, which is speculated to facilitate HCV replication (53). AGO binding of vRNA was observed at multiple regions of viral genome and particularly clustered at the HCV 5′ UTR miR-122 sites, confirming AGO engagement of vRNA in a replication-dependent manner (52). In a subsequent study with recombinant virus, pharmacologic inhibition and a single-cell reporter assay further elucidated the ability of HCV RNA replication to derepress host cell miR-122 production that can lead to potential oncogenesis after viral infection (52).

Inside the virus particle, HITS-CLIP has also been demonstrated to be a reliable method to characterize interactions between the RNA genome and structural proteins. This was well illustrated for RNA–NC interactions in influenza A virus (IAV) by Lee *et al*. (54). Infected cells were crosslinked, lysed, nuclease-digested, and IP-ed with nucleocapsid antibodies for different IAV strains. HITS-CLIP sequence data revealed distinctive patterns of binding loci for each strain but that consistently favored G-rich and U-poor RNA regions. In a follow-up study, Le Sage *et al*. (55) extended the HITS-CLIP in-virion binding profiles to other strains of influenza virus and found that both IAV and influenza B virus exhibited “nonuniform and nonrandom” binding patterns between vRNA and NC and that there is uneven distribution of NC-binding sites among different vRNA segments.

In spite of the power of HITS-CLIP, several limitations still apply. While the high energy of short wavelength UV radiation (UVB, UVC) can efficiently stabilize RNA–protein complexes (usually within several seconds), UV radiation may also lead to mutagenesis in the target of interest (56, 57, 58). Additionally, unprotected RNA is prone to fragmentation after excessive UV irradiation and oxidation thereafter (59, 60). Although HITS-CLIP is readily applied in cell culture, short wavelength UV is limited in its penetration depth, which typically cannot exceed the second layer of epidermis (stratum lucidum) (61). This prevents HITS-CLIP’s application to complex tissues or organs. It is also important to note that during short wavelength UV radiation, protein-RNA crosslinks are not exclusive (61, 62, 63) and extensive RNA-RNA crosslinking can also occur (64, 65), which can interfere with the interpretation of true RNA–protein interactions. During downstream analysis, bioinformatic tools have been developed to maximize the specificity and sensitivity of HITS-CLIP data (reviewed in (66, 67)). Nonetheless, HITS-CLIP is relatively coarse in terms of revealing nucleotide-resolution maps of RBP-binding sites (usually at a resolution of 30–60 nts). This is partially due to the lack of complete biophysical knowledge regarding UVB/UVC crosslinking specificity and their amino acid/nucleotide preferences (28). New bioinformatic tools allow for the detection of single nucleotide deletions and transitions in HITS-CLIP data (68). However, it remains complex to discern signals from background due to mutations occurring with different efficiencies for each nucleotide (69). Other technical artifacts such as mis-priming during reverse transcription (in the form of overrepresentation of sequences complementary to the primer) remain to be challenges for HITS-CLIP (70).

### PAR-CLIP

PAR-CLIP provides a different path to high-throughput discovery of RNA–protein interactions (23). Based on the same principle as CLIP, PAR-CLIP integrates a significant variation through the use of photoactivatable nucleoside analogs. These enhance UV crosslinking by reducing the required excitation energy and also increasing crosslinking efficiency (23, 71, 72, 73). Analogs such as 5-azidouracil, 8-azidoadenine, 8-azidoguanine, 4-thiouracil, 5-bromouracil, 5-iodouracil, and 5-iodocytosine have all been successfully used (reviewed in (71)). Among these, thionucleobases such as 4-thiouracil (4SU) and 6-thioguanosine (6SG) have gained popularity for a number of reasons including the following: (1) sulfur is only 0.45 Å larger than oxygen (71, 74), allowing minimum structure perturbation; (2) 4SU or 6SG can be supplemented in cell culture at high concentrations without obvious cytotoxic effect (23, 72, 73, 75); (3) the sulfur substitution allows for UV excitation around 330 to 365 nm, avoiding the 260 nm excitation wavelength of native uracil and minimizing unwanted photochemistry and/or photodamage (71, 74); (4) free radicals of the thio-group can greatly enhance crosslinking efficiency and crosslinking yield can reach up to 90% (76); and (5) importantly, the 4SU/6SG incorporated RNA can lead to specific base mismatches during reverse transcription (U-G, and G-T) (77, 78, 79). This latter point is potentially due to alterations in hydrogen bond donor/acceptor within a base pair as a result of the crosslinked peptide adduct (80) (Fig. 2*B*). These crosslink-specific mutations (U-C or G-A transitions) enable high-throughput screening for the precise nucleotide at the RNA-protein crosslink and therefore engaged in RNA–protein interactions.

PAR-CLIP takes advantage of thionucleobase-enhanced crosslinking with the following basic scheme (illustrated in Fig. 1): (1) modified nucleotides such as 4SU are supplemented in cell culture and converted to 4SUTP, which are subsequently incorporated into newly synthesized RNA; (2) 4SU-labeled cells are washed and crosslinked with long wavelength UV irradiation (typically UVA at 365 nm); (3) lysed cells are treated with RNase T1 and IPed on magnetic beads with an antibody specific to the protein of interest; (4) the enriched RNA–protein complex is then treated with RNase T1 again to ensure the removal of uncrosslinked or unprotected RNA, which is followed by radiolabeling of crosslinked RNA; (5) the recovered RNA-RBP from SDS-PAGE EMSA is then digested by proteinase K and the purified RNA-polypeptide complex is ready for reverse transcription and NGS library preparation (23, 81, 82). In the final sequence data, the RBP-interacting 4SU or 6SG is determined by U-to-C or G-to-A transitions, respectively. This mutation profile informs the crosslinking position and ultimately the RNA sites that interact with protein. Compared to HITS-CLIP, which only reveals the approximate binding site position, PAR-CLIP therefore provides a nucleotide-resolution map of RNA–RBP interaction sites.

PAR-CLIP provides a dependable method for identifying RNA/miRNA targets of important cellular proteins, such as Argonaut 2, embryonic lethal abnormal vision (ELAV) protein, pumilio homolog 2 (PUM2), insulin-like growth factor proteins (23, 83), as well as identifying actively transcribed tRNA genes by targeting pre-tRNA binding protein La (lupus antigen, a ubiquitous pre-tRNA–binding protein) (84). In virus research, like HITS-CLIP, PAR-CLIP was quickly adapted to identify the miRNA targets of human herpesviruses including KSHV and EBV. AGO PAR-CLIP uses 4SU to crosslink AGO with interacting miRNAs and mRNAs. Thousands of viral miRNA/mRNA targets were revealed to interact with AGO in latent cell lines infected with either KSHV or EBV (69, 85, 86). Among the discovered miRNA interaction sites, 7% were EBV miRNAs in infected lymphoblastoid cells lines (85), and 30%-27% were derived from KSHV miRNAs in infected BCBL-1 and primary effusion lymphoma cell line BC-1 (86), which is drastically different from the HITS-CLIP study of KSHV-infected BC-3 cells, where ∼83% of miRNA reads were of viral origin (42). In comparison to previous studies (42, 46, 47, 69), both PAR-CLIP and HITS-CLIP demonstrated great reliability in determining miRNA targets, such as EBNA2, LMP1, and BHRF1 for EBV and miR-K10a, -K10b, and miR-142-3p for KSHV (69). As for AGO-mRNA targets, the number of genes regulated by viral or host miRNAs were found to be limited in both EBV and KSHV, while instead the majority of mRNA matches (of KSHV-encoded miRNAs) were targets within the host transcriptome (85, 86). This is expected as viral replication cycles are minimal in the selected latent cell lines (69). Among the AGO mRNA targets characterized by PAR-CLIP, cellular pathways such as transcription regulation, intracellular signaling and transportation, protein localization, MAPKKK (Mitogen Activated Protein kinase kinase kinase), were found to be consistently influenced by both EBV and KSHV miRNAs (86). This suggests a functional similarity between KSHV and EBV miRNAs, despite their evolutionary distance.

PAR-CLIP has also been extensively utilized in HIV research. The HIV-1 Gag polyprotein contains viral structural proteins that coordinate numerous features of the viral lifecycle including genome selection, intracellular genome trafficking, virion assembly, budding, and maturation (reviewed in (87, 88)). This indicates that there are extensive interactions between Gag and numerous host and vRNAs at each of these different stages. Kutluay *et al*. (89, 90) used PAR-CLIP to enrich Gag–RNA complexes form both cells and virions to investigate the global Gag-RNA interactome during and after Gag-orchestrated genome assembly. This uncovered a surprising and drastic shift in profiles of Gag-interacting RNAs during HIV-1 intracellular virion assembly (90). The cytosolic monomeric Gag–RNA complexes occurred at discrete sequences within both the 5′ leader and 3′ Rev Response Element (RRE) of the HIV genomic RNA, suggesting these sites may be spatially adjacent (90). This was contrasted by the membrane-bound, oligomeric Gag-RNAs, which bound a range of sites across viral genome. Such dynamic RNA-binding properties of Gag are also observed during the viral maturation process. Mature virions, which comprise proteolyzed Gag, exhibited a Gag-RNA profile similar to that of the cytosolic fraction, whereas the immature virion exhibited significant similarity to membrane-bound Gag-RNA profile. In addition to vRNA, HIV-1 also packages cellular RNAs (91). Motif enrichment analysis (92) uncovered that cytoplasmic Gag binds to GU-rich cellular RNAs while membrane-bound Gag favors A-rich motifs (90). In the same study, individual domains of Gag were also investigated with PAR-CLIP to characterize RNA-binding specificity. The matrix domain (MA) of Gag was found to be devoid of vRNA but exclusively interacted with host tRNA (90). Interestingly, such MA–tRNA interactions showed strong preference for specific tRNA anti-codons. Specific tRNAs such as Glu^CTC^, Glu^TTC^, and Gly^GCC^ were bound up to 100-fold more frequently than others (90). In contrast, NC was found to be primarily crosslinked to vRNA and largely resembled the interaction profile of full-length Gag. The detailed dissection of Gag domains, in combination with PAR-CLIP, addressed the question of whether MA can interact with viral or cellular RNAs (93). Together, Gag PAR-CLIP offered a valuable approach to determine the reciprocal and dynamic relationship between HIV-1 Gag and RNA in the context of membrane association during virion genesis.

HIV-1 integrase (IN) is a multi-domain enzyme and one of the cleaved products of Pol polyprotein (94). IN mediates the integration of viral DNA into host chromosomes following the production of double-stranded proviral DNA from reverse transcription (95, 96). In addition to integration, IN has long been suggested to coordinate viral replication and virion maturation. Mutated IN can lead to the eccentric “exile” of ribonucleoprotein complexes (RNPs) outside of capsid shell and ultimately impairs virion maturation (97, 98, 99, 100, 101). Using PAR-CLIP, Kessl *et al*. identified IN-binding RNA targets in virions (102). IN showed strong binding preference for the trans-activation response (TAR) element (103) and RRE, but not for the packaging element ψ, suggesting IN and NC have both shared and unique roles in HIV-1 genome assembly and particle maturation (102). *In vitro* biochemical experiments and mass spectrometry–based protein footprinting (104) were conducted to validate IN-RRE/IN-TAR binding and to evaluate their affinities with deletions/changes of structural elements. The quinoline-based allosteric HIV-1 integrase inhibitors are a class of anti-HIV agents that bind noncatalytic sites of IN, preventing IN–vRNA interactions (105). In one study, Madison *et al*. (106) used allosteric HIV-1 integrase inhibitors (107) to promote eccentric maturation of HIV-1 and applied PAR-CLIP (alternatively named “CLIP-seq” in this study) to reveal conservation of NC–vRNA interaction sites, despite vRNA being mislocated outside the capsid shell. Together, PAR-CLIP unveiled the correlation of IN–vRNA interactions and particle maturation.

In addition to important HIV-1 viral proteins, PAR-CLIP has been used to investigate several HIV-1-related cellular factors, antiviral proteins, and RNA modifications (reviewed in (108)). For example, APOBEC3 (A3) is a family of cytidine deaminases that can suppress a broad range of viruses, including HIV-1 (109, 110). PAR-CLIP has been used to uncover the preferential binding of A3 proteins to cellular and virion vRNAs, suggesting binding specificity is influenced by nucleotide composition (G-rich and/or A-rich) instead of sequence (111). In a following study, PAR-CLIP revealed the virion-encapsidated human A3H haplotype II (huA3H) protein favors interaction with short RNA duplexes (7 nucleotides) (112). Another host protein related to HIV-1 pathogenesis is the Zinc finger antiviral protein (ZAP). ZAP represents another class of antiviral host factors targeting a broad range of viruses by promoting the degradation of viral mRNAs (113). PAR-CLIP provided evidence that ZAP binds highly specifically to CpG dinucleotides (114). This allows for ZAP to differentially target viral but not host mRNA, as the latter is typically depleted of frequent CpG dinucleotides (115). It is also interesting to note that some have speculated that HIV has similarly began evolving to evade this mechanism by altering dinucleotide content (116, 117).

In host cells, DNA methylation of provirus promoter regions has been shown to regulate HIV-1 latency and transcription activation (118). In addition, RNA methylation has recently been recognized as an important factor in HIV-1 RNA metabolism and replication (119). Methylation of adenosine at the N6 position (m6A) is the most prevalent mRNA modification, which are ‘*read*’ by the cytoplasmic YTH domain family (YTHDF) proteins (120). One study used PAR-CLIP to enrich YTHDF–vRNA complexes and unveiled that m6A modifications were clustered at HIV-1 3′ UTR exclusively (121). Interestingly, another study applied HITS-CLIP instead of PAR-CLIP (122) and determined that binding sites of YTHDF1–3 proteins are located in both 5′ and 3′ UTRs. This discrepancy provides an interesting comparison of the two methods and suggests that HITS-CLIP allows for a more permissive and therefore sensitive detection of protein-binding sites, while PAR-CLIP provides a more stringent but specific readout.

PAR-CLIP applications in virus studies are far beyond the above-mentioned human herpesviruses and HIV. Influenza virus, flavivirus, and alphavirus research have all benefited from PAR-CLIP and its extensions. We listed more selected applications in Table 1.

Similar to HITS-CLIP, PAR-CLIP also has several intrinsic limitations. The introduction of a nucleoside analog to nascent RNA transcripts or vRNAs limits PAR-CLIP applications within the scope of cell culture and *in vitro* systems instead of *in vivo* research. Photoactivatable nucleoside-induced cytotoxicity (123), as well as other cellular stress and inhibitory effects (124), should also be critically evaluated for each cell line or experimental model. The concentration and uptake efficiency of analogs also require optimization. Typically, only one analog is applied in PAR-CLIP experiment. This dictates that the observable interacting sites are limited to the crosslink events at that particular nucleotide (*e.g.*, only U crosslinks can be revealed with 4SU supplement in cultured cells). Although it is possible to supplement with a different analog in parallel experiment (*e.g.*, 6SG), they each exhibit different crosslink efficiencies and signal intensities (23). Similarly, thio/UVA–induced crosslinks only favor reactive amino acid side chains (mainly phenylalanine, tyrosine, and tryptophan, while lysine and cysteine can also be crosslinked to a lesser extent) (77, 79), hence certain RNA–protein interaction lacking these favorable amino acids may be missed. The long and technical experimental procedures of PAR-CLIP (Fig. 1) also demand a large quantity of starting material (usually starting between 10^8^ and 10^9^ cells) (81). Additional concerns such as antibody specificity, IP efficiency, as well as whether a crosslink can interfere with antibody binding, should also be considered for PAR-CLIP, as with HITS-CLIP and other IP-based techniques.

### vPAR-CL

**Figure 3:**
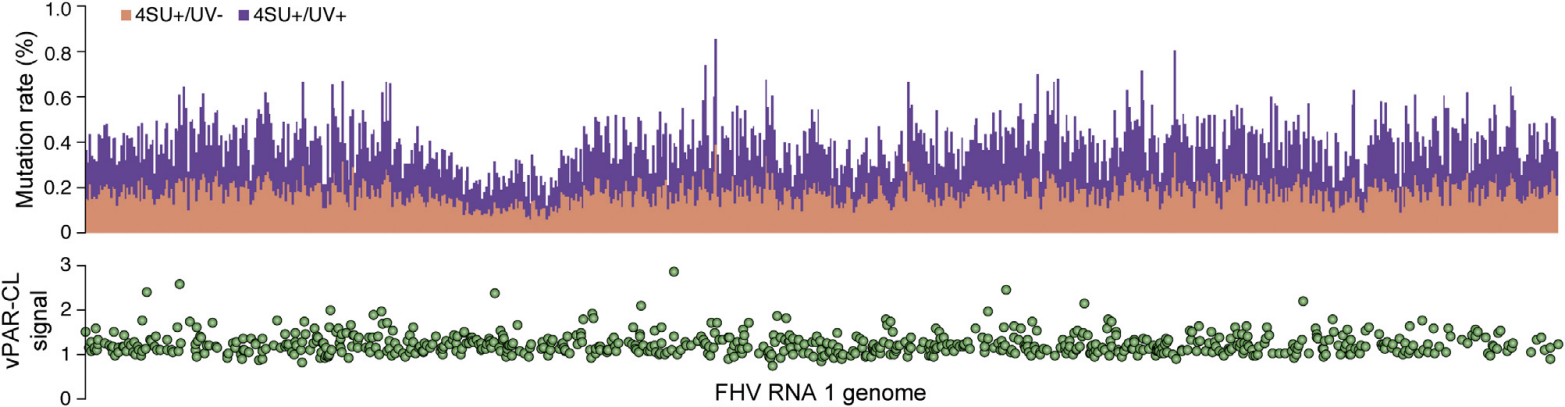
**Example of vPAR-CL signals across full-length RNA1 genome of FHV.** The U-C transition rates (*upper*) were compared between cross-linked virus (4SU+/UV+) and non-cross-linked control virus (4SU+/UV−) to yield vPAR-CL signals (*lower*), which represents the fold change of U–C transitions. Adapted from the study by Zhou and Routh (24).

vPAR-CL represents a technique that is different from other CLIP-based methods. vPAR-CL is specifically designed to investigate the *in virio* RNA–protein interactions of an RNA virus (24). In contrast to the complex cellular micro-environment, vPAR-CL takes advantages of the unique and highly confined enclosure of an assembled virus, which only consists of few well-defined components (*e.g.*, vRNA and capsid). In return, vPAR-CL obviates the time- and resource-consuming IP process but instead relies on the standard purification of the given virus (24). vPAR-CL starts in an analogous fashion to PAR-CLIP (Fig. 1), whereas a photo-activatable nucleoside analog (*e.g.*, 4SU) is supplemented to cell culture during virus infection to label newly synthesized vRNA genomes. After cell lysis, the virus particles are purified (typically with PEG precipitation and/or sucrose gradient ultracentrifugation) and RNase treated to remove any copurified unpackaged viral or host RNAs. The intact, 4SU-containing viruses are crosslinked with 365 nm UV and then are subjected to proteinase K digestion, RNA extraction, reverse transcription, and NGS library construction. In comparison to PAR-CLIP (23, 81, 82), vPAR-CL avoids the need for labor-intensive procedures such as IP of RBP, dephosphorylation, radio-labeling, SDS-PAGE and electro-elution, and therefore significantly shortens the time frame of experiments and the requirement of starting materials. When coupled with the fragmentation-free NGS technology such as ClickSeq (125, 126), vPAR-CL can yield results with less than 2 μg of viral particles (equivalent to 250 ng of vRNA in the case of Flock House virus (FHV)) (24), while typical PAR-CLIP starts with a large number of cells (*e.g.*, 10^8^ cells or approximately 10–50 15 cm cell culture plates for HEK293 cells) (81, 82). Removing the IP in vPAR-CL mitigates the bias generated due to specificity of antibodies and efficiency of IP and the associated procedures. This also prevents any potential interference of IP that is caused by RNA-protein crosslinking. Without any artificial enrichment, vPAR-CL largely minimizes the background noise, as any random, nonspecific crosslinked signals and intrinsic viral mutations will be diluted, while specific and consistent RNA-capsid interactions can readily be discerned (24). An important and novel feature of vPAR-CL is derived from the fact that all purified vRNA is sequenced (rather than just the IP-enriched crosslinked fragments) from both crosslinked RNA (4SU+/UV+) and noncrosslinked control (4SU+/UV-). As a result, vPAR-CL directly compares the U-to-C transition rates between conditions. This eliminates intrinsic mutational events (127) from interaction site interpretation (24) (Fig. 3) but also provides a ratiometric value for RNA-crosslinking, allowing for both the identification of RNA regions that are bound to RBPs in a conserved and structured manner as well as providing information on which sites do not interact with RBPs.

vPAR-CL has been demonstrated with FHV to reveal highly conserved RNA-capsid interactions across the entire encapsidated viral genome. This suggests a structural tropism of the RNA inside virion (24) that likely reflects the dodecahedral RNA cage previously observed for FHV RNA in x-ray and cryo-EM studies (128). The distribution of RNA-capsid interaction clusters also suggests that the packaged FHV genome has multiple conserved RNA-capsid–binding sites. This indicates that the genome packaging mechanism of FHV could resemble that of other +ssRNA viruses (16). Combined with dimethyl sulfate mutational profiling with sequencing (129), it was also uncovered that FHV RNA-capsid interactions are enriched in dsRNA regions, and the disruption of base pairing at these vPAR-CL sites interfered with viral fitness (24).

vPAR-CL represents a rapid and labor-friendly method to specifically study *in virio* RNA–protein interaction sites. Both vPAR-CL and PAR-CLIP utilize photo-activatable nucleoside analogs to achieve efficient crosslinking and investigate nucleotide-resolution interactions sites. Therefore, vPAR-CL shares the same biochemical limitations as PAR-CLIP related to thio-based photo-crosslinking. Additionally, due to the lack of enrichment of crosslinked 4SU-containing RNA–protein complexes, the incorporation rate of 4SU is critical in vPAR-CL experiments, which determines whether the vPAR-CL signal can stand out above the background. This requires optimization on 4SU dosage and virus harvest timing. vPAR-CL is demonstrated to be reliable to resolve the protein-interaction sites in icosahedral particles such as FHV, whose RNA genome is in direct contact with single protein component-the capsid. It remains an open question whether vPAR-CL can be adapted to other enveloped viruses such as flavivirus, in which the vRNA genome may be in contact with multiple protein components (17).

### Other CLIP-derived methods

There are many techniques derived from the same rationale as CLIP but differ in downstream procedures and applications to identify specific protein–RNA interactions in cells, viruses, and *in vitro* (reviewed in (130, 131, 132)). Nonetheless, the principles behind these protocols remain largely the same, as do many of the steps necessary to conduct these experiments and analyze the resulting sequencing data. Here, we will only provide a brief overview to compare and contrast a handful of these CLIP-derived methods based on their hallmarks.

In individual nucleotide resolution CLIP (iCLIP) (133), crosslinked RNA-RBP sites are identified *via* cDNA chain termination at the introduced covalent bonds during reverse transcription, which is expected to occur in more than 80% cases (133). This contrasts HITS-CLIP which typically characterizes the approximate crosslinking sites after cDNA read-through. To capture these truncated cDNA, the reverse transcription primer contains both the 3′ and 5′ adapters for sequencing. The transcribed cDNA is then intramolecularly circularized. This, importantly, allows for nucleotide resolution mapping of the interaction site, which is located one nucleotide upstream of the truncation site.

Infrared-CLIP (134) utilizes antibody-conjugated beads to IP RNA–RBP complex followed by on-bead RNase digestion to maximize retained RNA fragments. This is followed by the ligation of an IR800-biotin adapter to the 3′ end of IP-ed RNAs to avoid the standard radioisotope labeling (of HITS-CLIP and PAR-CLIP) at the 5′ ends of RNA molecules. The infrared-biotin adapter not only prevents the inconsistent autoradiography signals due to radioisotope decay but also largely reduces the time required for protein–RNA complex visualization with equivalent efficiency.

In simplified CLIP (135), after IP, crosslinked RNA is biotin labeled and subsequently visualized *via* streptavidin-horseradish peroxidase to avoid radiolabeling. This is followed by proteinase K digestion and polyadenylation of RNA to allow reverse transcription with a modified oligo-d(T) primer. This obviates the ineffective RNA ligation of sequencing adapters, allows for low input materials during reverse transcription, and omits size-selection of cDNA products. Therefore, as the name suggests, simplified CLIP presents a simplified and efficient procedure compared to traditional HITS-CLIP.

Enhanced CLIP (136) builds upon the iCLIP protocol but instead incorporates two separate adapter ligation steps: (1) after IP, the enriched RNA is dephosphorylated and the first ssRNA adapter is ligated to the 3′ end of crosslinked RNA. This first adapter contains an “in-line-barcode” to enable the pooling of similar molecular weighted samples from multiple experiments; (2) The second ssDNA adapter, which contains a random nucleotide sequence (a random-mer), is ligated to the 3′ end of cDNA after RT. This ssDNA adapter serves to preserve the single-nucleotide resolution of terminated cDNA reads. The random nucleotide sequence of the second adapter (also commonly referred as unique molecular identifier) allows for demultiplexing and to determine whether identical sequenced reads represent two unique RNA fragments (bearing different random-mer sequences) or the PCR duplicates of the same RNA fragments (bearing the same random-mer sequences). Together, enhanced CLIP provides an approach to greatly shorten the hands-on time of typical CLIP-based techniques and to reduce nonspecific RNA–protein interaction artifacts and PCR bias during data interpretation.

In terms of virus research, we list examples of these CLIP-derived methods that have been used to study protein–RNA interactions in response to viral infections (Table 1).

## Chemical crosslinking

Although photo- (UV-) crosslinking methods offer reliable solutions to characterize direct RNA-protein contacts with nucleotide or near-nucleotide resolution, methods such as HITS-CLIP, PAR-CLIP, vPAR-CL, and other similar technologies are only effective for *in vitro*, in virion, and cell culture studies. When it comes to a whole tissue or whole organism scale, UV-light often falls short in providing a viable crosslinking solution, due to its deficiency in evenly penetrating dense and complex tissues. Instead, crosslinking methods using chemicals such as formaldehyde are widely used due to their ability to permeate different tissues.

Formaldehyde elicits crosslinking of a broad range of biomolecules. Between protein and nucleotides, formaldehyde first reacts with protein nucleophiles to yield an imine (Schiff Base), whose subsequent interaction with amino groups of DNA/RNA bases results in covalent crosslinking (Fig. 2*C*) (reviewed in (21)). In comparison to “zero-distance” photo-crosslinking, the additional carbon of formaldehyde extends the crosslinkable range to ∼2 to 3 Å (21) (2 N-C bonds between nucleic acid and amino acid) but still allows for crosslinking of macromolecules within close proximity (137, 138). It has been suggested that the numerous formaldehyde aggregates in commercial products can further extend the distance-spanning capability to much greater distances (21, 139). Therefore, formaldehyde is able to crosslink and capture certain RNA–protein interactions that are otherwise excluded from UV crosslinking (140, 141). An important feature of formaldehyde crosslinking is its reversibility. The heat and salt conditions allowing for formaldehyde crosslink reversal have been well characterized (138, 142, 143) and are critical in ChIP/ChIP-seq experiments (144).

PIP-seq (141, 145, 146) was therefore developed, which combines crosslinking, RNase-mediated protein footprinting, and high-throughput nuclease-sensitivity sequencing assays (147, 148). Although PIP-seq can also utilize UV to crosslink RNA-RBP, formaldehyde remains the most common reagent in this application. In PIP-seq, formaldehyde-crosslinked cells are lysed and total cellular RNA–protein complexes are separated into two pools. Pool 1 (experimental group) undergoes RNase digestion and subsequent heat reversal to enrich for protein-bound RNA footprints (crosslinked and hence protected from RNase digestion). Pool 2 (control group) is first subjected to proteinase K treatment and subsequent RNase digestion. This is followed by heat reversal of formaldehyde crosslinks. The recovered RNAs from both pools are used as input for NGS library synthesis. Comparison of the read coverage across the whole host transcriptome is drawn to identify protein-protected RNA sites, which is represented by a differential signal between pool 1 and pool 2 (141, 145, 146). The use of ssRNA- or dsRNA-specific RNases allows identification of ssRNA or dsRNA sites that are associated with proteins (145). PIP-seq has been applied in human cell (HeLa) (141) and plant cell (145, 149, 150) transcriptomes and identified numerous novel RPB-binding motifs, including enriched RBP-reacting polymorphisms that are associated with diseases (141).

PIP-seq is not accompanied with any conventional IP methods to allow specific enrichment of RBPs of interest. Unlike vPAR-CL which is only applied in virions with highly selected RNA/protein components, PIP-seq instead searches the entire transcriptomic length in a complex cellular or *in vivo* setting. This reveals the global state of protein-bound RNA sites, without informing on the counterpart (RBP). To address this, ‘RNA:protein immunoprecipitation in tandem (RIPiT)-Seq’ can be performed (151). RIPiT-Seq resembles a chimeric form of PIP-seq and RIP (Native RNA IP)-seq, which combines formaldehyde crosslinking, RNase footprinting, and IP to allow discovery of RNA-binding sites of RBP of interest. Of note, in RIPiT-Seq, the RBP of interest is typically additionally labeled (*e.g.*, FLAG-tag). After formaldehyde crosslinking, two sequential IP steps (with anti-FLAG and anti-RBP, respectively) are involved and separated by RNase treatment to generate RNP footprints. This is followed by crosslink reversal, RNA extraction, dephosphorylation, size selection, and NGS library construction (151). As a major advantage of RIPiT-Seq, the two sequential IPs (which is enabled by additional FLAG tag) significantly deplete intracellular RNA species (*e.g.*, rRNA fragments, tRNAs), which therefore improves signal-to-noise ratio in the downstream NGS assays.

Although formaldehyde is commonly regarded as a more efficient and powerful crosslinker to UV irradiation, formaldehyde readily induces protein-protein crosslinking, which can interfere with the specificity of identification of desired RNA–protein interaction as well as yielding indirect signal (149). It is also important to recognize the formaldehyde aggregates in commercial formalin solution (21, 139) can potentially introduce significant amount of long-range, nonspecific multi-component crosslinks in biological samples. The required sample-by-sample optimization of crosslinking conditions (to avoid over-crosslink) and the RNA thermo-damage during crosslink reversal also remain as challenges of PIP-seq method and other formaldehyde-based approaches.

## Affinity-capture methods and proteomics

Besides IP, affinity-capture (152) provides an alternative solution to specifically purifying protein contents of interest as well as to enrich crosslinked RNA sequences. There are numerous adaptations of HITS-CLIP or PAR-CLIP, in which IP was replaced with affinity-capture methods. One innovative application that combines affinity-capture and NGS is proximity-CLIP, which utilizes the unique abilities of engineered ascorbic acid peroxidase protein 2 (APEX2) to biotinylate proximal endogenous proteins with biotin-phenol moieties (153, 154, 155). In proximity-CLIP (156), APEX2 is fused with a localization element to specifically target a subcellular compartment, and cells are infused with 4SU. APEX2 is activated by biotin-phenol and subsequent hydrogen peroxide addition to covalently tag proteins of proximity with biotin. This is followed by 4SU-induced UVA crosslinking (as described for PAR-CLIP) to capture nascent transcripts interacting with biotinylated proteins. Compartment-specific RNPs and proteins are then captured by streptavidin affinity chromatography. Quite uniquely, proximity-CLIP allows for concurrent compartment-specific analysis of both proteome (*via* mass spectrometry) and interacting RNAs (*via* RNA-seq) (156). A similar technology, APEX-seq (157, 158, 159), is based on the same rationale and provides yet another solution to depict both the proteome and its interacting RNA profiles with specificity to a certain cellular compartment. In contrast to the 4SU-induced crosslinking of proximity-CLIP, APEX-seq typically crosslinks RNA to proteins *via* formaldehyde (159) or even bypasses crosslinking altogether (157, 158). In virus research, APEX-seq data was referenced in a study investigating localizing signals in SARS-CoV-2 RNA, predicting that both genomic and subgenomic vRNAs are localized to the host mitochondria and nucleolus (160).

As a recent development, VIR-CLASP (VIRal Crosslinking And Solid-phase Purification) also presents a method that is designed to investigate the protein components of the vRNA-cellular RBP interactions (161). In VIR-CLASP, in a similar fashion to vPAR-CL (24), cells are 4SU labeled and infected with virus of choice, such as chikungunya virus (161). This is followed by UVA crosslinking and cell lysis. The denatured RNA–protein complexes are then recovered with solid-phase purification with solid phase reversible immobilization (162) beads to selectively enrich for nucleic acids. RNA is digested with nucleases and the crosslinked protein components are identified by LC–MS/MS. In addition to chikungunya virus, VIR-CLASP also successfully identified host proteins interacting with viral genomes of IAV (163), Zika virus (164), and SARS-CoV-2 (165). Notably, similar techniques have been applied previously to study the mRNA-binding proteins in mammalian cell lines (166), with the main difference being the use of oligo(dT) beads to harvest crosslinked RNA–RBP complexes. Interestingly, in this study, the use of 4SU-induced crosslinking and mass spectrometry for protein identification is informally termed “PAR-CL”, which is not to be confused with vPAR-CL.

There are also numerous RNA-centric methods that utilize affinity-capture to investigate RNA–protein interactions. For example, RNA Bind-n-Seq (167, 168) is based on the same principle of RIP-seq where no crosslink was induced. However, in RNA Bind-n-Seq, streptavidin-binding peptide tag was used to purify the targeted protein with streptavidin magnetic beads. Similarly, crosslinking and affinity purification (iCLAP) (169) purifies double-tagged (Strep/His) RBPs using streptavidin beads.

## Non-crosslinking methods

### RIP-seq

Native RNA immunoprecipitation (RIP) was first applied to isolate and purify proteins interacting with the XIST RNA that controls X chromosome inactivation (170, 171). RIP quickly partnered with NGS to form RIP-seq to allow high-throughput screening of Polycomb repressive complex 2 (PRC2) interacting RNAs in embryonic stem cells (172). The rationale of RIP-seq is largely similar to HITS-CLIP, with the main difference of the lack of crosslinking (171, 172). Instead, RIP-seq relies on the native high affinity between certain RNAs and RBPs to withstand the subsequent IP and purification. In RIP-seq, cell or nuclear lysates are directly subjected to specific antibody binding and bead pull-down. Strongly bound RNAs are retained after extraction and NGS libraries and bioinformatics assays are conducted thereafter (172, 173). RIP-seq is demonstrated to be a versatile method of transcriptomic miRNA/mRNA-RBP profiling in the fields of stem cell research, RNA epigenetics, and alternative splicing (172, 174, 175, 176). Alternatively to NGS, RIP has also been combined with microarray analysis to yield another high-throughput application: RIP-Chip (177).

In virus research, Lichinchi *et al*. combined m^6^A IP and RIP-seq (MeRIP-seq) to study the HIV-1 induced m^6^A increase in both host and viral mRNAs and uncovered the connection between HIV-1 RRE methylation and nuclear export efficiency of RNA (178). Similarly, a recent study combined m^6^A-RIP-seq and m^6^A-PAR-CLIP (miCLIP) (179) to reveal eight putative m^6^A sites in the SARS-CoV-2 genome (180). Importantly, single-nucleotide variants associated with these identified m^6^A sites allowed the phylogenetic clustering of specific US epidemic strains of SARS-CoV-2. In silkworms infected with *Bombyx mori* nucleopolyhedrovirus (BmNPV), Nie *et al*. conducted AGO2 RIP-seq and identified numerous small noncoding RNAs, including highly enriched rRNA-derived fragments (181). With EBV, AGO2-RIP-seq depicted the viral and cellular miRNA landscape in diffuse large B-cell lymphoma cell lines (182) and lymphoblastoid cell lines (183). Another important EBV protein is Epstein-Barr nuclear antigen 1 (EBNA1), which plays a vital role in viral replication and the partitioning of viral genomic DNA during latent viral infection but it has also been shown to alter splicing of host mRNAs (184). EBNA1-RIP-seq clearly showed that EBNA1 bound specific cellular targets such certain mRNAs and noncoding RNA (184). Interestingly, the discovered EBNA1-RIP-seq targets did not include any gene whose splicing was modulated by EBNA1, suggesting that EBNA1’s ability to modulate splicing does not require direct interaction between EBNA1 and target RNAs (184).

In RIP-seq, the obviation of crosslinking largely shortens the experimental procedure and reduces the requirement of starting material. However, the exclusion of crosslinking also limits the application of RIP-seq. The requirement for strong interactions within an RNA–RBP complex that can withstand purification procedures is unguaranteed and the IP of noncrosslinked RNA inevitably precludes stringent washing during purification that would remove contaminants. Similarly, RNase digestion must be controlled carefully in RIP-seq. RIP-based techniques are typically suitable for stable RNP, such as those interacting with noncoding RNAs (185). RIP therefore provides a suboptimal solution for the study of transient or less stable RNA–protein interactions. Furthermore, false positive signals can arise from the reassociation of RNA (particularly mRNA) and proteins in the lysate (185, 186, 187, 188).

### SHAPE/DMS flexibility probing

Selective 2′-hydroxyl acylation analyzed by primer extension (SHAPE) and dimethyl sulfate (DMS) chemistries have been extensively used as RNA secondary structural probing methods, due to their extraordinary abilities to methylate flexible nucleotides in biological samples (SHAPE methylates 2′-OH of RNA ribose while DMS methylates N1 of adenine and N3 of cytosine) (189, 190, 191, 192, 193). More recently, SHAPE or DMS probing has been empowered by high-throughput NGS and advanced bioinformatics to generate mutational profiles (MaPs) to offer incredible depth and single nucleotide resolution to transcriptomic scale RNA structure discovery (129, 194, 195, 196, 197, 198). As great as their power in ssRNA probing, it is often overlooked that SHAPE and DMS are, at their core, nucleotide flexibility probes. Both structural (*e.g.*, base pairing) and functional restrictions (*e.g.*, RNA–protein interaction, DNA nucleosome formation) can preclude methylation and allow unveiling of the structural/functional relations. Indeed, Smola *et al*. (197) used SHAPE-MaP to investigate intracellular RNA–protein interactions: SHAPE chemical 1M7 was applied under native conditions to living cells as well as extracted and refolded RNAs. The differences in SHAPE reactivity can hence be compared as the *ex vivo* RNA samples are deproteinized and lack numerous RNA–protein interactions compared to *in cellulo*. Similar to PIP-seq (141), SHAPE-MaP and related chemical probing methods provide a high-throughput transcriptomic scan of RNA–RBP interaction sites, with little prerequisite for existing knowledge of involved proteins or RNAs. In addition, Kenyon *et al*. also combined SHAPE-MaP with photo-crosslinking to fully take advantage of both worlds (199). XL-SHAPE (crosslink and SHAPE) utilizes SHAPE reagent N-methylisatoic anhydride in conjunction with 254 nm UV irradiation to concurrently capture RNA structural changes as well as protein-binding sites. With *in vitro* crosslinking, XL-SHAPE successfully identified the RNA-interacting site of HIV-1 Tat/TAR (200) complex and Gag-binding site on HIV-1 leader sequence. This was further complimented by differential SHAPE reactivities of bound/unbound RNAs (199). Similar “function-to-structure” investigation has also been conducted on FHV, in which vPAR-CL and dimethyl sulfate mutational profiling with sequencing were used to identify clustered RNA–capsid interactions on dsRNA (24).

## Perspective

Virus research has been fueled by rapidly emerging high-throughput and quantitative tools to study RNA–protein interactions. Since the development of HITS-CLIP, RNA research as well as the RNA virus field has evolved to transcriptomic and genomic scale studies. It is apparent that the NGS-enabled RNA–protein interaction technologies are an ever-growing field and that more and more such techniques will continue to be applied in an expanding range of viral and biological systems. It is inevitable that many of these technologies have been adapted or developed to study pressing issues such as the ongoing SARS-CoV-2 pandemic.

**Figure 4:**
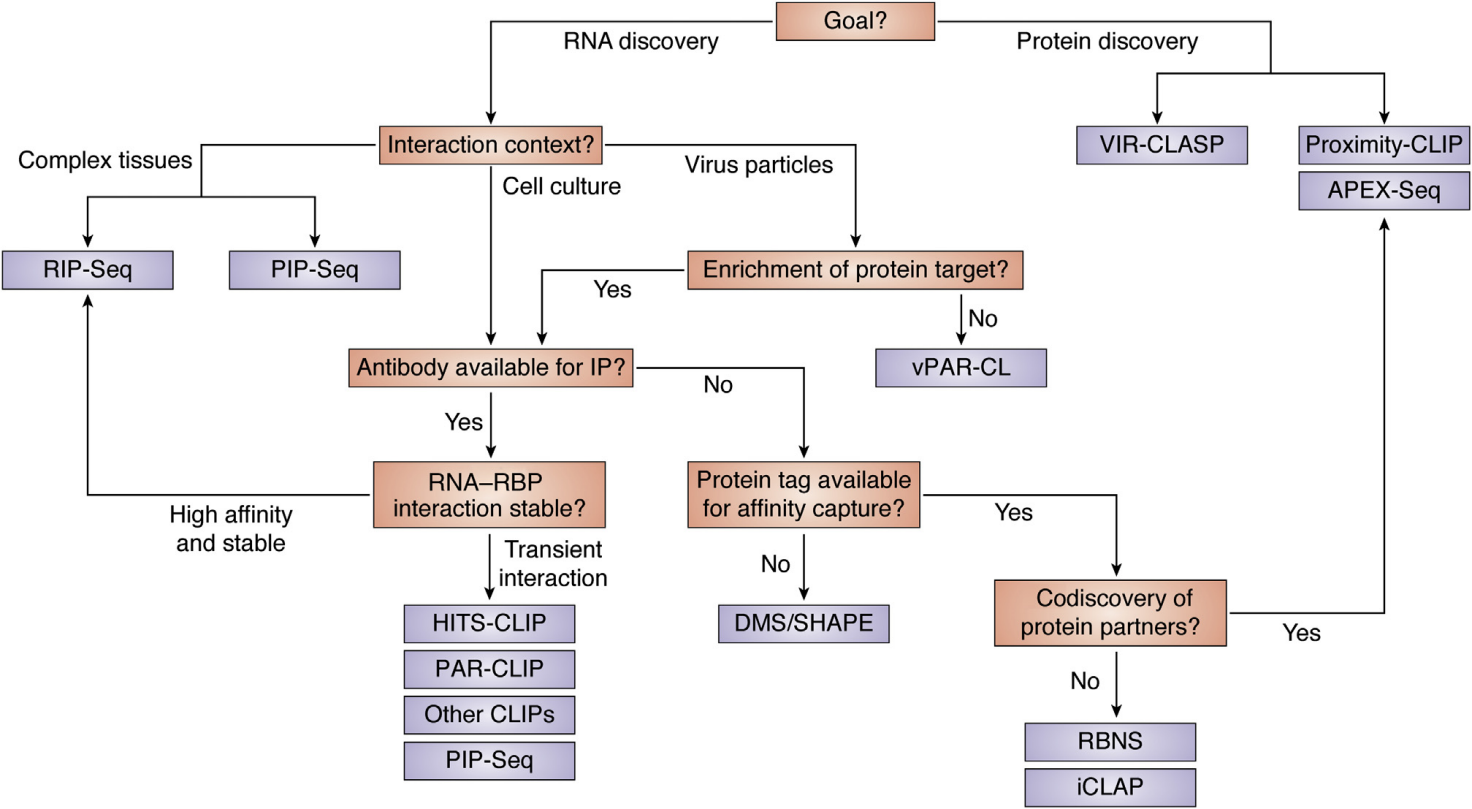
**A quick reference flowchart based on different methods’ discovery goals, starting materials, and procedural requirements.**

With such a great availability of NGS-enhanced technologies to investigate RNA–protein interactions, it could be overwhelming to determine the most appropriate scenarios to apply each technique. We hereby provide a quick reference flowchart (Fig. 4), in which we categorized the above-mentioned approaches based on their discovery goals (RNA-centric or protein-centric discoveries), their starting materials, and procedural requirement (*e.g.*, whether antibody is available for IP). This flowchart is intended for brief guidance, while many other factors should be considered during experimental design.

Certainly, many challenges remain. Although novel methods such as vPAR-CL reduce experimental burden, many techniques are still labor-intensive and require handling of hazardous materials such as radioisotopes. Both technological and computational advances are necessary to improve RNA-binding site discovery to have a higher sensitivity, lower bias, and better specificity. It is also challenging to adapt these methods to applications in more complex scenarios such as in animal models or higher plants. Finally, as many techniques only focus on end-point analysis, it is important but remains difficult to understand how the RNA–protein interactions and the associated regulatory networks dynamically change at different timescales. This is especially important to virus research as vRNA genomes encounter a myriad of protein partners throughout their replication cycles.

It is also tempting to cross-compare different platforms for their reliability, sensitivity, and the biological relevance of the discovered sites. However, it remains challenging to draw such conclusions, as different experimentalists will inevitably employ different cell lines, culturing conditions, virus strains, infection methods to address their own research need. Numerous crosslinking methods, enzymes (83), sequencing adapters (201), and bioinformatic pipelines (67, 202) can all dictate the final output of experiments. To the best of our knowledge, there is no systemic study available thus far to compare different approaches in detail. Nevertheless, a few studies managed to provide a glimpse at how certain methods overlapped or contrasted with others, in their own specific experimental scenarios. In one forementioned example, two studies tackled HIV-1 mRNA methylation sites with PAR-CLIP and HITS-CLIP (122). PAR-CLIP revealed (121) the exclusively enriched m6A sites at 3′ UTR whereas HITS-CLIP identified sites at both 5′ and 3′ UTRs (122). The exact nucleotide sequences of identified sites in these two studies are also different. In another study (83), both HITS-CLIP and PAR-CLIP were used with the same bioinformatic framework to investigate RNA-binding sites of HuR protein (203). It is shown that each method delivered reproducible results with similar correlation coefficient between replicates (83). However, there was no direct cross-comparison between HITS-CLIP and PAR-CLIP in regard to the proportion of overlapped discoveries. These limited examples suggest divergencies of the different approaches: each highlights its own advantages and limitations; each has its own focus (*e.g.*, HITS-CLIP focuses on a wide range of binding sites, while PAR-CLIP focuses on higher specificity of the binding sites); each will discover different sets of RBP-binding sites on a given transcriptome or genome. It also demonstrates that the NGS and bioinformatic discoveries resulting from any of these approaches should be considered as a highly efficient hypothesis-generating approach, which ultimately necessitate experimental validation.

Looking ahead, several rapidly growing new technologies also help depict a bright picture of virus RNA–protein interaction research. For example, single-cell RNA sequencing allows for comprehensive understanding of an individual cell in the context of its original microenvironment. Additionally, Oxford Nanopore Technologies’ long-read nanopore sequencing is advantageous in generating long and continuous single-molecule sequencing reads, which can effectively cover the entire virus genome to understand the coevolution of distant virus mutations and other features (204). Furthermore, nanopore sequencing can directly sequence RNA in its native state, allowing for the detection and resolution of modified RNA bases which may be extended to detect peptide adducts generated due to RNA-protein crosslinking. Altogether, it is foreseeable that both single-cell RNA sequencing and Nanopore sequencing, among other new technologies, will further lead the vRNA-protein interaction research to new and exciting avenues in the near future.

## Conflict of interest

The authors declare that they have no conflicts of interest with the contents of this article.

## Footnotes

† Interestingly, HITS-CLIP was renamed “CLIP-Seq” in a study of RbFOX2 splicing regulator targets in human embryonic stem cells (219). Notably, this alternative term triggered certain confusions, as a number of studies also referred such term to a collection of similar technologies that combine CLIP and NGS, such as PAR-CLIP (81). For clarity, in this review, CLIP-Seq refers to the alternative naming of HITS-CLIP, and other NGS techniques utilizing CLIP will be discussed separately.
